# The influence of selenium concentration and variations in selenoprotein genes on the *CHEK2*-dependent cancers

**DOI:** 10.1186/1897-4287-13-S1-A13

**Published:** 2015-09-09

**Authors:** Satish Gupta, Magdalena Muszynska, Katarzyna Jaworska-Bieniek, Grzegorz Sukiennicki, Katarzyna Kaczmarek, Anna Jakubowska, Jan Lubinski

**Affiliations:** 1 Department of Genetics and Pathology, International Hereditary Cancer Center, Pomeranian Medical University, Szczecin, Poland; 2 Read Gene SA, Szczecin, Poland

## Introduction

Studies in the Polish population provided evidence that four founder mutations in *CHEK2* gene (1100delC, IVS2+1G>A, I157T, del5395) are associated with increased risk of cancers including breast, prostate, colon, kidney and thyroid. The substantial variability in cancer penetrance in mutation carriers can probably be explained by the influence of other genetic and/or environmental factors. Selenium is an essential component of several major metabolic pathways, including the antioxidant defense system and the immune system. It has been suggested that selenium and variations in selected selenoprotein genes may be risk factors for several cancer types.

## Aim

The aim of study was to check if Se concentration and alteration in genes coding selenoproteins can be associated with cancer risk in *CHEK2* mutation carriers and unselected patients.

## Material and method

Biological samples (blood, serum, plasma) and clinical information have been collected from 826 *CHEK2* mutation carriers - 670 healthy individuals and 156 cancer cases - including breast, gastrointestinal tract (colon, intestine, pancreas, stomach, gallbladder), respiratory system (lung, larynx), prostate, kidney, ovarian and others.

In the above groups cancer patients have been matched to unaffected individuals in ratio 1:2 or 1:1. The matching included *CHEK2* mutation type, sex, year of birth, smoking, number and location of cancer among Iº relatives.

In all matched individuals Se concentration in serum/plasma have been determined using ICP-MS and 4 SNPs in selenoprotein genes *GPX1 (rs1050450), GPX4 (rs713041), TXNRD2 (rs1139793)* and *SEP15 (rs5845)* have been analyzed using Taqman probes.

To assess the cancer risk in relation to Se level and/or studied alterations in selenoprotein genes the odds ratio have been calculated using conditional logistic regression for matched groups. In tested groups the cancer risk in relation to Se concentration was analyzed in quartiles. All statistical analyses were performed using R software.

## Results

In the group of the matched *CHEK2* carriers mean selenium level was significantly lower in cancer patients than in healthy controls: 73.2µg/l vs. 78.5µg/l (p=0.002). We found that the cancer risk decreased with increased Se concentration: individuals in IV^th^ quartile (>86.28 µg/l) in comparison to those in I^st^ quartile (<65.81µg/l) have ~2-times lower cancer risk (OR=0.46; p=0.01). We also observed an association of *CHEK2* associated cancer risk depending on variants in selenoprotein genes. The data analysis indicates modest association of rs5845 in *SEP15* with breast, rs1050450 in *GPX1* with prostate cancer and rs713041 in *GPX4* with lung/larynx cancer risk. Influence of Se level on breast, prostate, lung and larynx cancer risk may be dependent on variants in selenoprotein genes.

## Conclusions

Our analyses clearly indicate that selenium can be a modifier of cancer risk in *CHEK2* mutation carriers. The effect of selenium level in blood serum on cancer risk may be dependent upon genotypes in selenoprotein genes.

